# Does Mother Provide? Re‐Examination of Maternal and Embryonic Microbiomes of *Diploptera punctata*

**DOI:** 10.1002/ece3.74504

**Published:** 2026-10-12

**Authors:** Brennan Jonas, Victoria Arrowsmith, Paul Ayayee

**Affiliations:** ^1^ Biology Department University of Nebraska, Omaha Omaha Nebraska USA

**Keywords:** *Blattabacterium*, brood sac, *Diploptera punctata*, gut microbiomes, viviparous

## Abstract

The cockroach endosymbiont *Blattabacterium cuenotii* plays a central role in host nutrition throughout development, particularly during embryogenesis in the viviparous cockroach *Diploptera punctata* . However, the presence and potential functional relevance of non‐blattabacterial taxa in developing embryos of this species remain unclear. Previous studies of maternal and embryonic microbiomes in *D. punctata* , based on Illumina 16S rRNA sequencing, have yielded inconsistent findings, partly due to differences in how endosymbiotic sequences were treated. Here, we reanalyzed microbiome data from two studies, with and without Blattabacterial reads, to reevaluate the presence and composition of non‐blattabacterial taxa in embryonic microbiomes. Embryonic microbial communities (1) differed significantly from each other between datasets and (2) from associated maternal gut communities, suggesting that environmental (maternal, dietary, and laboratory) and sequencing factors may influence their acquisition and composition rather than reflecting a stable, consistently transmitted microbiome. Nevertheless, the detection of non‐blattabacterial taxa in embryonic microbiomes suggests the possible acquisition of non‐endosymbiotic bacteria by developing embryos. The functions of these microbes are speculated, but were not explicitly tested in this study and remain to be verified. Overall, our findings suggest that although non‐endosymbiotic embryonic bacteria may be environmentally acquired and variable, their biological relevance remains unresolved and cannot be determined from 16S amplicon data alone. This highlights the importance of investigating acquired non‐endosymbiotic bacteria and their potential functional roles in the development of viviparous cockroaches.

## Graphical abstract

This figure summarizes the compositional differences in the non‐blattabacterial microbiomes of embryonic and adult females of the viviparous, *Diploptera punctata*. This shows the non‐negligible presence and possibly maternal sources of these bacterial taxa in developing embryos.

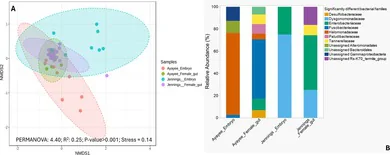

## 1 Introduction

Vertical transmission, including transovarial transmission, of obligate bacterial endosymbionts in insects is critical to host nutrition, defense, development, and growth across diverse taxa (Bruijning et al. 2022; Maeda et al. 2024; Wierz et al. 2021). Insects also harbor more variable gut‐associated microbiota that similarly contribute to host development and fitness (Douglas 2009; Kaufman et al. 2000; Yun et al. 2014). Endosymbionts typically reside intracellularly within specialized tissues, maintain persistent host associations, are maternally transmitted, possess reduced genomes, and perform specialized functions. In contrast, gut microbiota generally inhabit the gut lumen, exhibit dynamic community membership, are acquired primarily from the environment and social interactions, retain larger and more flexible genomes, and perform diverse functions (Mazel et al. 2024; Michalik et al. 2023; Shropshire et al. 2020).

In cockroaches (Blattodea), the vertically transmitted endosymbiont *Blattabacterium cuenotii*, housed in bacteriocytes within the fat body of embryonic and post‐embryonic stages, recycles nitrogenous waste and synthesizes essential amino acids for the host (Bandi et al. 1995; Noda et al. 2020; Sabree et al. 2009; Silva et al. 2024). Cockroaches also harbor diverse gut microbial associates that complement *Blattabacterium* functions and, in some cases, perform functions unavailable to the endosymbiont (Ayayee et al. 2016, 2020; Bertino‐Grimaldi et al. 2013; Carrasco et al. 2014, 2015; Jahnes et al. 2019; Jahnes and Sabree 2020; Lampert et al. 2019; Schauer et al. 2012; Silva et al. 2024; Tinker and Ottesen 2016, 2021). These gut associates are primarily acquired horizontally through coprophagy of conspecific frass by newly hatched nymphs, contributing to the establishment of stable, context‐dependent gut communities (Jahnes et al. 2019; Justinn et al. 2021; Kopanic Jr and Schal 1999; Kopanic Jr et al. 2001). However, evidence suggests that cockroach eggs and newly hatched nymphs may harbor non‐*Blattabacterium* taxa before coprophagy. Non‐blattabacterial taxa have been detected in surface‐sterilized German cockroach ootheca (Carrasco et al. 2014) and in first‐instar brown‐banded cockroaches after removal of *Blattabacterium* reads following 16S amplicon sequencing (Guse and Pietri 2024). Although these observations could reflect sequencing or processing artifacts, they suggest that some gut‐associated microbes are acquired prior to post‐hatching coprophagy in cockroach species.

In the viviparous cockroach *Diploptera punctata* , embryos develop within the brood sac and receive a nutritive milk secretion throughout development (Stay and Coop 1973; Williford et al. 2004). Two studies have characterized the microbiomes of gravid females and their embryos but reached contrasting conclusions regarding the potential role of non‐blattabacterial bacteria in embryonic development. Ayayee et al. (2017) found several non‐blattabacterial taxa enriched in embryos relative to associated females and proposed that these bacteria may complement nutritional deficiencies in the milk by synthesizing methionine and tryptophan, which are scarce in the secretion and cannot be synthesized by the endosymbiont *Blattabacterium* (Ingram et al. 1977; Kambhampati et al. 2013; Sabree et al. 2009; Williford et al. 2004). Although direct experimental evidence is lacking, genomic, metatranscriptomic, and stable‐isotope data support the feasibility of such complementary non‐endosymbiotic/gut bacterial metabolic pathways (Dukes et al. 2023; Pereira and Berry 2017). In contrast, Jennings et al. (2019) concluded there were no prominent non‐blattabacterial taxa (nor potential functions) associated with embryonic development. A key methodological difference between these studies is that Ayayee et al. (2017) specifically excluded *Blattabacterium* from their microbiome analyses (since they are not technically gut microbial associates), whereas Jennings et al. (2019) retained blattabacterial reads. The discrepancy between the two studies might also be due to laboratory, dietary, or methodological variation. Thus, whether non‐blattabacterial microbes are detected or are associated with developing *D. punctata* embryos remains unresolved. Consequently, any speculated or hypothesized potential functions of the associated non‐blattabacterial taxa remain unexamined. Resolving this discrepancy is necessary to determine whether a non‐endosymbiotic embryonic microbiome is present and, if so, its biological relevance to embryonic nutrition and development. Although endosymbionts are not technically gut microbes, they can dominate 16S amplicon datasets from insect hosts, substantially reducing the resolution of lower‐abundance bacterial taxa and potentially obscuring ecological patterns in community composition, diversity, and abundance (Basit et al. 2025; Guse and Pietri 2024; Simhadri et al. 2017; Li et al. 2022; Mazel et al. 2024; Sonett et al. 2024). Thus, while retaining endosymbiont reads is appropriate for studies of host–endosymbiont dynamics, their inclusion is less appropriate when the objective is to characterize the broader, non‐endosymbiotic microbial community.

We therefore reanalyzed the combined 16S amplicon datasets from Ayayee et al. (2017) and Jennings et al. (2019), both with and without *Blattabacterium* reads. Because *Blattabacterium* comprised most raw reads in both datasets, we excluded these reads to minimize compositional bias and improve detection of low‐abundance non‐blattabacterial taxa. Although this approach resulted in substantially fewer retained sequences, it provided greater resolution of the microbial communities relevant to our ecological questions. Specifically, our primary objective was to assess the presence, potential inheritance, and composition of non‐blattabacterial microbes associated with developing *Diploptera punctata* embryos, with a secondary objective of evaluating their potential functional capabilities using the limited taxonomy‐based inference approach. The resulting increase in non‐blattabacterial sequence after removal of *Blattabacterium* reads supports more effective evaluation of potential embryonic acquisition of these microbial associates.

## 2 Materials and Methods

### 2.1 Amplicon Data Acquisition, Curation, and Processing

Raw 16S amplicon read data generated using the 515f (GTGYCAGCMGCCGCGGTAA) and 806r (GGACTACNVGGGTWTCTAAT) universal primers targeting the V4 (V4‐V5) hypervariable region of the 16S rDNA gene from both studies were retrieved from the NIH/NCBI repository server using the Sequence Read Archive (SRA) server tool within the Galaxy platform (Version 26) (The Galaxy Community 2024) using the provided accession numbers from both studies (PRJNA522760 for the Jennings et al. 2019 dataset and PRJNA314839 for the Ayayee et al. 2017 dataset). The final combined dataset comprised 15 samples from the Ayayee_2017 publication (7 female gut and 8 embryo samples) and 42 samples from the Jennings_2019 publication (20 female gut and 22 embryo samples), yielding a total of 57 samples. The analysis of microbiome data from two separate studies required several steps, including data acquisition, quality control, and statistical analysis. Nymphal stages were excluded to ensure that only female gut and embryo samples were analyzed to assess embryonic acquisition.

Quality control and preprocessing of the fastq reads were performed using the DADA2 pipeline (Callahan et al. 2016) on the Galaxy platform (The Galaxy Community 2024). Raw fastq files were filtered and trimmed (Paired reads = TRUE, truncQ = 2, left and right Trim start = c(0,0), truncLen = c(150, 150), minLen = 20, Remove reads with more Ns (maxN) = 0, minQ = 0, expected errors, maxEE = c(2, 2), Discard reads matching PhiXgenome (rm.phix) = TRUE) to ensure uniform sequencing depth across both datasets. Chimeric sequences were identified and removed using the removeBimeraDenovo function in DADA2 in Galaxy, with the consensus method (Check samples independently for chimeras and make a consensus decision on each sequence variant). Standard read‐quality processing yielded a merged read of about 150 bp, ensuring substantial overlap of forward and reverse reads from both datasets, given the comparable MiSeq chemistries used (MiSeq v2 chemistry for the Ayayee _2017 dataset and MiSeq 2 × 300 bp chemistry for the Jennings_2019 dataset). This step was essential to maintain sequence quality without losing critical data or lowering quality scores. Subsequently, ASVs were generated, and ASV taxonomies were assigned using the SILVA 138.1 16S rRNA gene reference database (Quast et al. 2012). A classical ASV table was generated and used for downstream analyses with QIIME v.1.8 scripts (Caporaso et al. 2010; Kuczynski et al. 2012) and in MicrobiomeAnalyst (Lu et al. 2023) for comparative purposes.

### 2.2 Analyses of Datasets With *Blattabacterium* Reads

For analysis of datasets containing *Blattabacterium* reads, the final ASV table contained 11,693 ASVs after removing ASVs assigned to Eukaryota, mitochondria, chloroplasts, or unassigned at the Domain (Kingdom) level. We used this filtering level to retain all 57 samples and ensure consistent normalization before downstream analyses. Rarefaction is a standard and recommended approach for normalizing microbiome data (Cameron et al. 2021; Hong et al. 2022; Schloss 2023; Weiss et al. 2017). Therefore, all downstream analyses were performed using rarefied data, and no analyses were conducted on the non‐rarefied datasets. Based on the distribution of reads across samples, we selected a rarefaction depth of 3740 reads per sample. This depth sufficiently captured the species richness in the embryo samples, as indicated by the plateau of the rarefaction curves (Figure 1A). However, the rarefaction curves for adult female gut microbiome samples did not reach a plateau at this depth. All downstream analyses were performed using the resulting rarefied ASV table.

**FIGURE 1:**
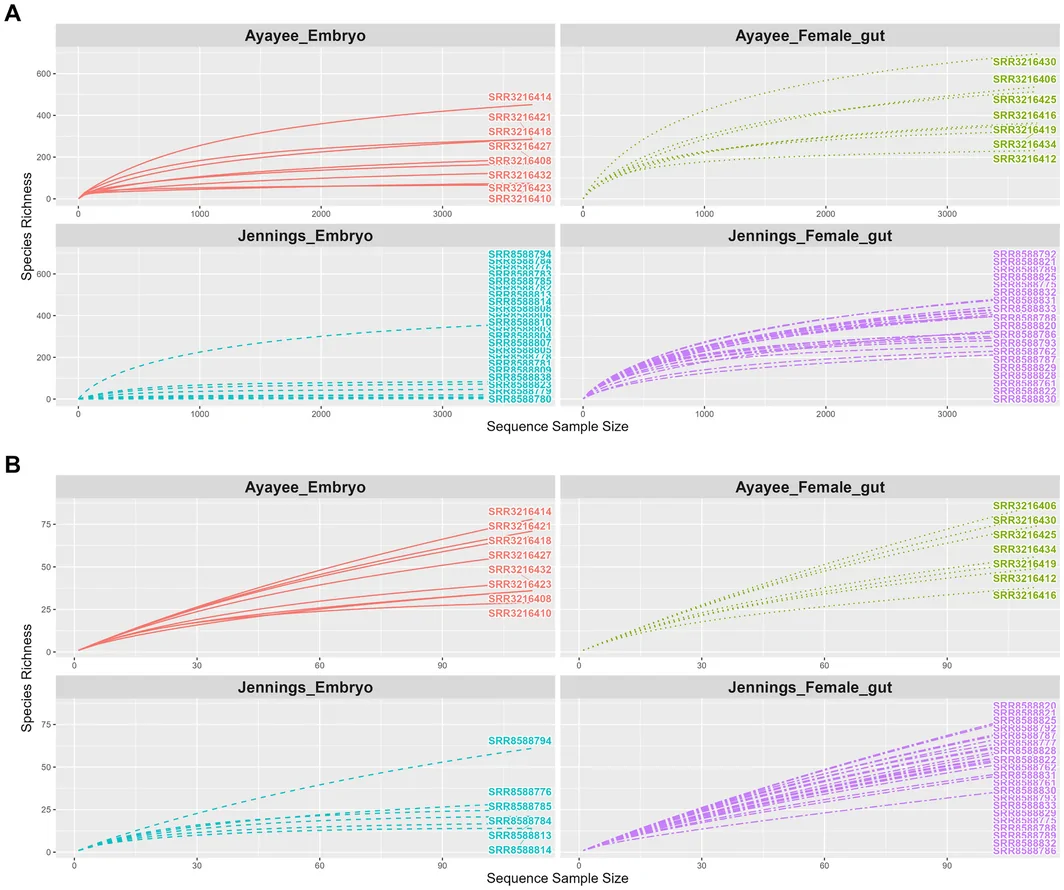
Rarefaction curves across the four sample categories show that (A) 3740 reads per sample adequately captured diversity in embryos and, to a lesser extent, adult female *Blattabacterium*‐retained microbiomes, whereas (B) 112 reads per sample constrained rarefaction in non‐*Blattabacterium* microbiomes across both embryos and adult females, but captured the expected low diversity.

### 2.3 Analyses of Datasets Without *Blattabacterium* Reads

After filtering ASVs classified as Eukaryota, mitochondria, chloroplasts, or unassigned at the Domain level, 11,469 ASVs remained. This filtering removed 224 ASVs assigned to *Blattabacterium* or *Blattabacteriaceae*. Based on sequencing‐depth distributions, samples with < 100 reads were excluded, and the remaining samples were rarefied to 112 reads per sample. This threshold balanced sequencing depth with sample retention given the substantial loss of endosymbiont‐derived reads, particularly from embryo samples. As a result, 14 embryo samples from the Jennings_2019 dataset were excluded (Figure 1B). All downstream analyses were conducted using the rarefied ASV table.

### 2.4 Diversity, Compositional, and Potential Function Analyses on Both Datasets

We assessed the microbiome richness of the gut microbiomes among the sample categories (Ayayee_Embryo, Ayayee_Female_gut, Jennings_Embryo, Jennings_Female_gut) using the Chao1 diversity index (Chao 1984), given its relevance for estimating total species richness (including rare species). We also assessed the evenness of gut microbiomes among sample categories using the Shannon index (Shannon 1948), given its sensitivity to the addition or loss of rare species and its ability to measure the even distribution of microbial taxa across categories. Compositional differences of the microbiomes were assessed using the Bray–Curtis dissimilarity distance matrix (Bray and Curtis 1957; McKnight et al. 2019), followed by permutational multivariate analysis of dispersion (PERMDISP) (Anderson and Walsh 2013) and variance (PERMANOVA) (Anderson 2017) on the generated distance matrix, followed by pairwise comparisons. Differentially abundant microbial taxa, highlighting calculated compositional differences among sample categories, were identified using the group_significance command in QIIME 1.9 (Caporaso et al. 2010; Kuczynski et al. 2012), which provides a Kruskal–Wallis test statistic and *p*‐value for each ASV, along with calculated abundance scores. The relative abundance of statistically significant ASVs among sample categories was plotted at the family level (for a clearer, less noisy overview). Potential microbiome functional capabilities were assessed using FAPROTAX with the respective rarefied ASV tables (Louca et al., 2016). This allows estimation of inferred microbial metabolic capabilities/functions based on the taxonomy of the ASVs in the tables. The resulting functional categories table was formatted as a classic OTU table and used as input to assess the differential abundance of potential functional categories using the group_significance command in QIIME. We used FAPROTAX because it provides a reasonable, limited, and well‐curated set of metabolic functional categories (compared with other similar amplicon‐based potential function inference tools that produce exhaustive lists of such potential functions). This was preferred over other methods, given the limitations of inferring functional potentials from an assigned taxonomy using the short 150 bp amplicon fragment of the ~1600 bp 16S rDNA gene. This was to avoid exhaustive speculation about potential functions and to highlight the limitations of this approach.

## 3 Results

### 3.1 Diversity and Compositional Analyses of Microbiomes With *Blattabacterium* Reads

Overall, we retained 62% of raw fastq reads from the combined dataset (Table S1) after quality processing. For ease of reporting and focus, we compared diversity and compositional analyses among the sample categories (Ayayee_Embryo, Ayayee_Female_gut, Jennings_Embryo, and Jennings_Female_gut). Overall, total bacterial and archaeal species count (abundant and rare species) based on the Chao 1 index differed among categories (*Kruskal–Wallis test‐statistic: 39.6; p > 0.001*) and was highest detected in the female gut samples (Ayayee_Female_gut, 528.75 ± 45.43, mean ± s.e., standard error, and Jennings_Female_gut, 394.23 ± 26.90), compared to associated embryo samples (Ayayee_Embryo, 247.85 ± 42.50 and Jennings_Embryo, 32.56 ± 25.60), with the least diversity detected in Jennings_Embryo samples (Figure 2A, Table 1A). Similarly, evenness differed among sample categories (*Kruskal–Wallis test‐ statistic: 42.42.20; p > 0.001*), with higher diversity and more balance microbiomes detected in the female gut samples (Ayayee_Female_gut, 5.2 ± 0.30 and Jennings_Female_gut, 4.1 ± 0.20) than embryo samples, with the Jennings_Embryo samples (0.41 ± 0.16) having the least balanced microbiome (Figure 2B, Table 1A). Assessment of community composition showed significant differences in dispersion among categories (*PERMDISP: F*_*(3,53)*_ *= 30.9; p = 0.001, number of permutations = 999*) and in variance (*PERMANOVA: F = 30.026; R* ^2^ *= 0.63; p = 0.001; Stress = 0.08*). This means that the four sample categories do not meet the assumptions of homogeneity; thus, the differences and associated community compositional shifts may be due to both true community shifts and differences in dispersion. Based on the PERMANOVA results, Ayayee_Female_gut, Ayayee_Embryo, and Jennings_Female_gut were more similar in composition compared to the Jennings_Embryo samples, although samples also differed significantly in composition following pairwise comparisons (Figure 3A, Table 1A). The clustering of the female gut microbiomes suggests comparable gut microbial composition among adult females (due to both fat body *Blattabacterium* reads and other gut microbiome members) following differential abundance analyses (Figure 3B). On the other hand, the clustering of Ayayee_Embryo samples with female gut samples is possibly driven mostly by the dominance of blattabacterial reads in the embryo samples, and to a lesser extent by the significant differential abundances of non‐blattabacterial reads (Figure 3B, Table S2A). The separation of the Jennings_Embryo samples from the other three sample categories may be underscored by the low abundances of less prominent non‐blattabacterial reads (Figure 3B, Table S2A).

**FIGURE 2:**
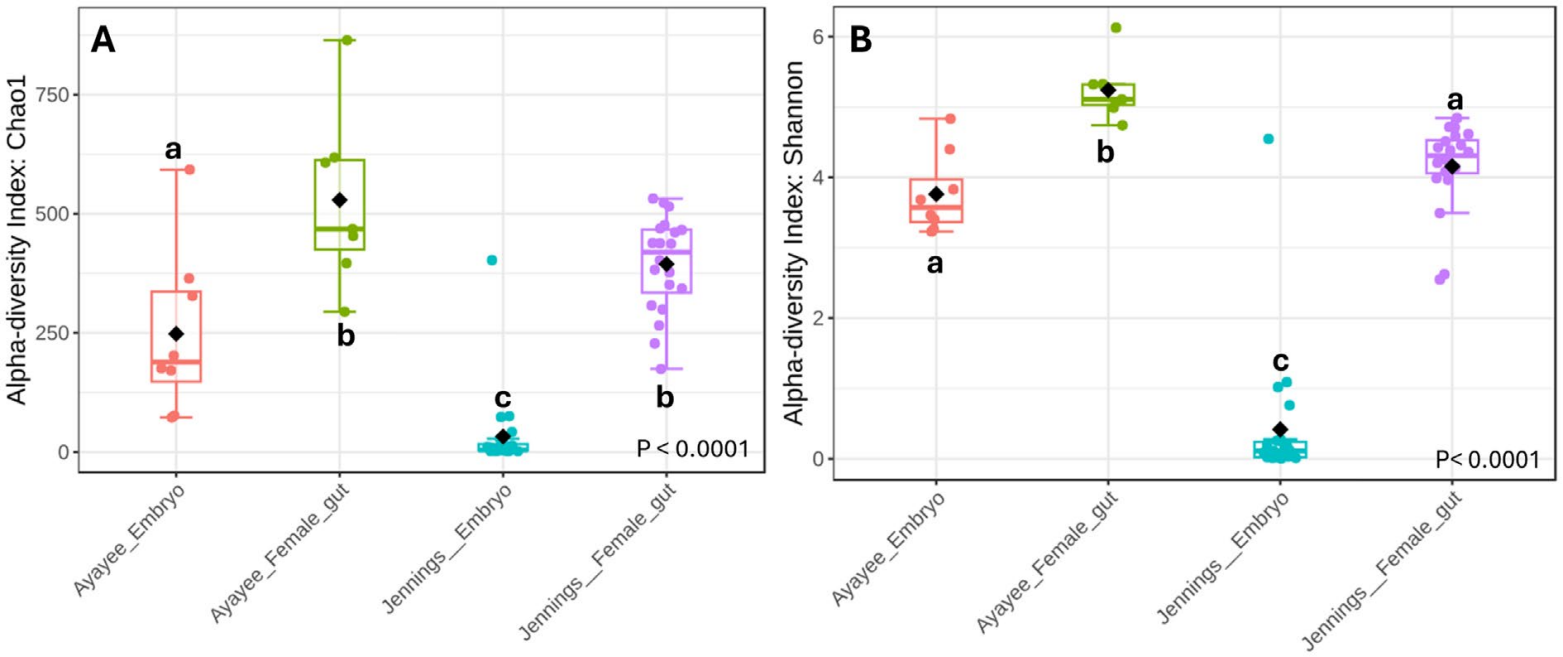
(A) Microbiome richness (Chao1) and (B) evenness (Shannon index) across the four sample categories following analysis with *Blattabacterium* reads. Different letters indicate significant pairwise differences (*p* = 0.05).

**TABLE 1:** Pairwise comparisons of alpha diversity and beta composition differences in (A) dataset with blattabacterial reads and (B) dataset without blattabacterial reads.

(A) With blattabacterial reads	Chao1		Shannon index	
Alpha diversity pairwise comparisons	Test‐statistic	*p*‐value	Test‐statistic	*p*‐value
Ayayee_Female_gut vs. Ayayee_Embryo	50	< 0.05	55	< 0.05
Ayayee_Female_gut vs. Jennings__Female_gut	100	0.1	139	< 0.05
Ayayee_Female_gut vs. Jennings__Embryo	152	< 0.05	154	< 0.05
Ayayee_Embryo vs. Jennings__Female_gut	34	< 0.05	45	0.08
Ayayee_Embryo vs. Jennings__Embryo	167	< 0.05	169	< 0.05
Jennings__Female_gut vs. Jennings__Embryo	430	< 0.05	425	< 0.05

		PERMANOVA Comparisons		
Compositional pairwise comparisons	*F*‐value	*R* ^2^	*p*‐value	
Ayayee_Female_gut vs. Ayayee_Embryo	13.222	0.50423	0.002	
Ayayee_Female_gut vs. Jennings__Female_gut	3.8103	0.13225	0.001	
Ayayee_Female_gut vs. Jennings__Embryo	60.409	0.69111	0.001	
Ayayee_Embryo vs. Jennings__Female_gut	15.923	0.37982	0.001	
Ayayee_Embryo vs. Jennings__Embryo	8.4186	0.23116	0.005	
Jennings__Female_gut vs. Jennings__Embryo	73.733	0.6483	0.001	

(B) Without blattabacterial reads	Chao1		Shannon index	
Alpha diversity pairwise comparisons	Test‐statistic	*p*‐value	Test‐statistic	*p*‐value
Ayayee_Female_gut vs. Ayayee_Embryo	39	0.23	36	0.4
Ayayee_Female_gut vs. Jennings__Female_gut	100	0.1	106	0.05
Ayayee_Female_gut vs. Jennings__Embryo	54	< 0.05	54	< 0.05
Ayayee_Embryo vs. Jennings__Female_gut	69	0.60051	88	0.71
Ayayee_Embryo vs. Jennings__Embryo	59	< 0.05	59	< 0.05
Jennings__Female_gut vs. Jennings__Embryo	152	< 0.05	147	< 0.05

		PERMANOVA Comparisons		
Compositional pairwise comparisons	*F*‐value	*R* ^2^	*p*‐value	
Ayayee_Female_gut vs. Ayayee_Embryo	13.222	0.50423	0.002	
Ayayee_Female_gut vs. Jennings__Female_gut	3.8103	0.13225	0.001	
Ayayee_Female_gut vs. Jennings__Embryo	60.409	0.69111	0.001	
Ayayee_Embryo vs. Jennings__Female_gut	15.923	0.37982	0.001	
Ayayee_Embryo vs. Jennings__Embryo	8.4186	0.23116	0.005	
Jennings__Female_gut vs. Jennings__Embryo	73.733	0.6483	0.001	

**FIGURE 3:**
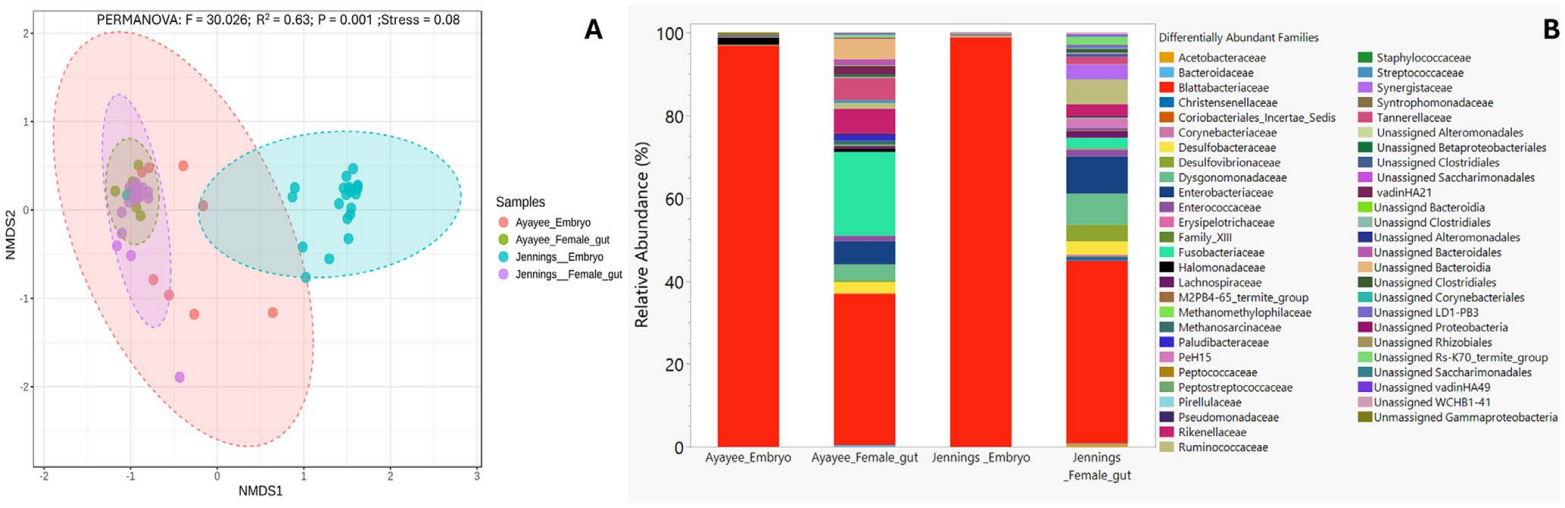
(A) NMDS showing significant differences in community composition across the four sample categories in the dataset with *Blattabacterium* reads (*PERMANOVA: F = 30.026; R* ^*2*^ *= 0.63; p = 0.001; Stress = 0.08*), and (B) The relative abundances of 55 differentially abundant bacterial ASVs (at the family level) out of a total of 11,693 ASVs in the dataset with *Blattabacterium* reads (*Kruskal–Wallis; FDR‐adjusted p‐value = 0.05*), likely driving differences in determined microbial community composition among categories.

We uncovered 23 differentially abundant microbial functional categories (*p < 0.05*) in our FAPROTAX assessment. Potential microbial functional categories chemoheterotrophy (aerobic and otherwise), fermentation, sulfate respiration, respiration of sulfur compounds, and ureolysis were differentially more abundant in female gut samples than in embryo samples (Figure 6A, Table S3A). Most of these potential categories can be attributed to members of the characterized gut microbiota. In contrast, ureolysis may be attributed to both free‐living gut microbiota and possibly *Blattabacterium* in the fat body, since it is known to recycle urea. Interestingly, the intracellular parasite functional category was differentially abundant in both female gut samples, with Jennings_Female_gut additionally showing an abundant mammal gut functional category (Figure 6A, Table S3A). The abundance of mammalian gut and intracellular parasites predicted potential functional categories in the Jennings_Female_gut samples possibly reflect the commensal nature of gut microbiota membership. These predicted functional categories are based on the reference database and ultimately the limitation of the taxonomical basis of how functional categories are inferred from this database using partial 16S amplicon data (Figure 6A, Table S3A).

With *Blattabacterium* reads present in the dataset, embryonic samples were dominated mostly by intracellular parasites and ureolysis‐potential functional categories (Figure 6A, Table S3A). As with the female gut samples, the intracellular parasite's potential functional category may reflect the presence of bacteria associated with taxa known to be intracellular parasites. The prominence of ureolysis can be attributed to either *Blattabacterium* or non‐blattabacterial reads capable of urea hydrolysis, underscoring *Blattabacterium*'s dominance in the embryo samples. Differentially abundant potential functional categories prominent in the Ayayee_Embryo samples (aerobic chemoheterotrophy, chemoheterotrophy, hydrocarbon degradation, and fermentation) are more comparable to those of adult female gut samples than to Jennings_Embryo samples, underscoring the higher abundance of non‐blattabacterial reads in the Ayayee_Embryo samples (Figure 6A, Table S3A).

### 3.2 Diversity and Compositional Analyses of Microbiomes Without *Blattabacterium* Reads

In the dataset without *Blattabacterium* reads, the total bacterial and archaeal species count (abundant and rare species) differed among categories (*Kruskal–Wallis test‐statistic: 17.40; p > 0.001*), and was highest in the female gut samples (Ayayee_Female_gut, 86.10 ± 6.52, and Jennings_Female_gut, 70.03 ± 3.86), with the Ayayee_Embryo samples intermediate between the female gut samples (**68.41 ± 6.10**), and Jennings_Embryo with the least richness (**33.35 ± 6.10**) (Figure 4A, Table 1B). The Jennings_Embryo samples had the least balanced microbiome (**2.71 ± 0.11**) (*Kruskal–Wallis test‐statistic: 16.60; p > 0.001*), relative to the Ayayee_Female_gut (**3.81 ± 0.12**), Ayayee_Embryo (**3.60 ± 0.11**), and Jennings_Female_gut (**3.57 ± 0.10**) (Figure 4B, Table 1B). Assessment of community composition showed significant differences in dispersion among categories (*PERMDISP: F*_*(3,39)*_ *= 1.32; p = 0.30, Number of permutations = 999*) and in variance (*PERMANOVA: 4.40; R* ^*2*^ *: 0.25; p‐value* > *0.001; Stress = 0.14*). All four sample categories met the assumptions of homogeneity, and the resulting differences reflect true community compositional shifts. Assessment indicated that Ayayee_Female_gut, Ayayee_Embryo, and Jennings_Female_gut were more similar in composition compared to the Jennings_Embryo samples (Figure 5A, Table 1B). There was notably greater overlap between the Jennings_Embryo samples and the other three sample categories than in the dataset containing *Blattabacterium* reads (Figure 5B, Table S2B). In contrast, the clustering of the female gut samples and the Ayayee_Embryo samples in this dataset suggests comparable differential abundances of non‐blattabacterial microbial composition despite differences in richness and evenness (Figure 5B, Table S2B).

**FIGURE 4:**
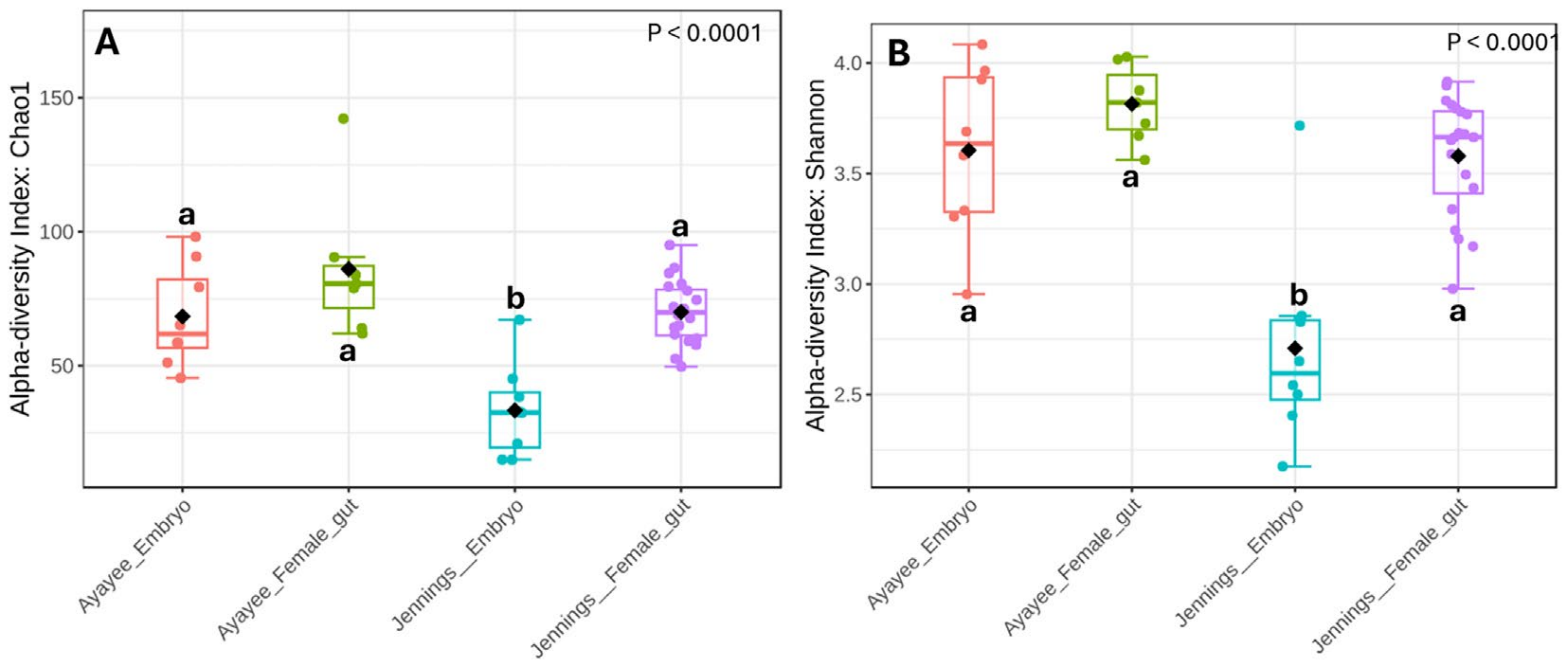
(A) Microbiome richness (Chao1) and (B) evenness (Shannon's index) across the four sample categories following analyses without *Blattabacterium reads*. Different letters indicate statistically significant differences following pairwise comparisons (*p = 0.05*).

**FIGURE 5:**
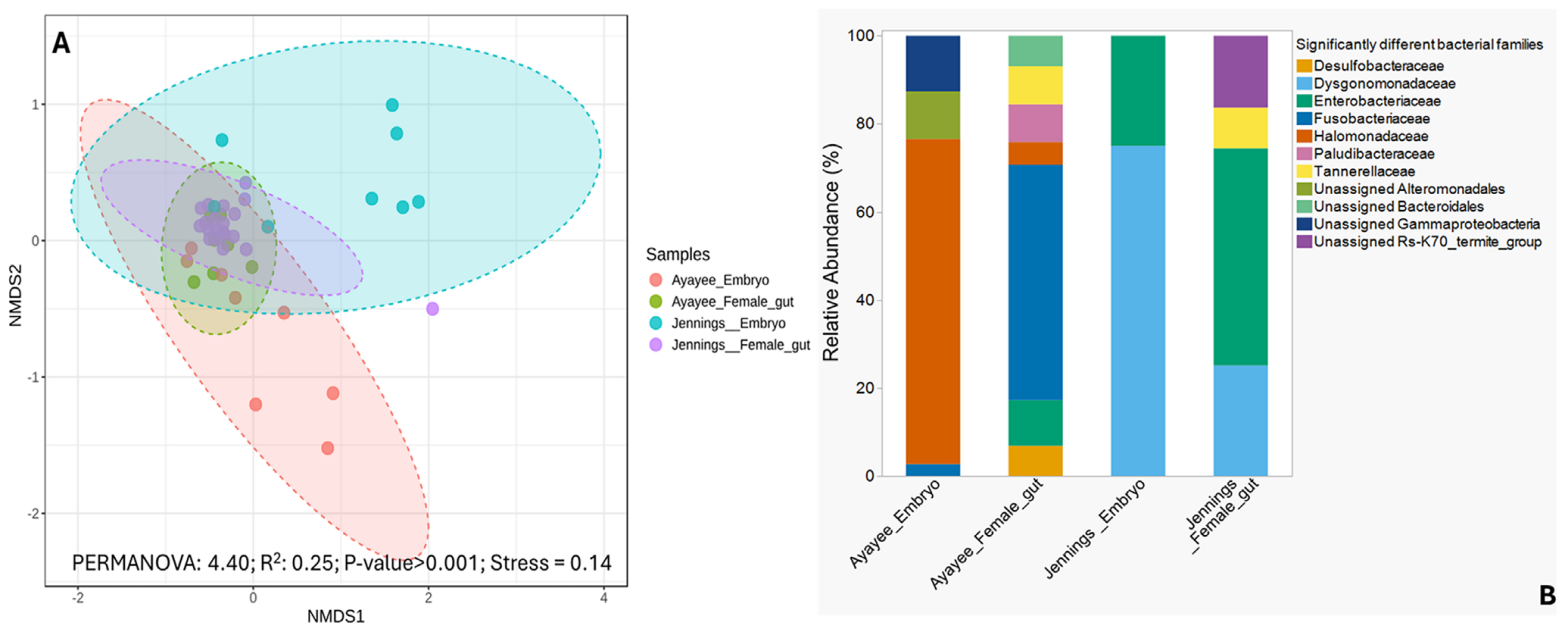
(A) NMDS showing significant differences in community composition across the four sample categories in the dataset without *Blattabacterium* reads (*PERMANOVA: F = 4.40; R* ^*2*^ *= 0.25; p‐value* > *0.001; Stress = 0.14*), and (B) The relative abundances of 11 differentially abundant bacterial ASVs (at the family level) out of a total of 11,469 ASVs in the dataset without *Blattabacterium* reads (*Kruskal–Wallis; FDR‐adjusted p‐value = 0.05*), likely driving differences in determined microbial community composition among categories.

Without *Blattabacterium* reads, we uncovered 10 differentially abundant microbial functional categories (*p < 0.05*) in our FAPROTAX assessment. Hydrocarbon degradation and methanogenesis (via methyl reduction) were prominent predicted potential microbial functional categories in the Ayayee_Female_gut samples, whereas human gut, human pathogens, human‐associated, ureolysis, and mammalian gut microbial potential functional categories were significantly more abundant in the Jennings_Female_gut samples (*p < 0.05*) (Figure 6B, Table S3B). Notably, these potential functional categories were absent in the Ayayee_Female_gut samples. However, aerobic chemoheterotrophy, sulfate respiration, and respiration of sulfur compounds were differentially abundant in both female gut samples (Figure 6B, Table S3B). Without *Blattabacterium* reads, aerobic chemoheterotrophy, sulfate respiration, and ureolysis were differentially abundant in Embryo samples. This possibly suggests non‐blattabacterial taxa capable of these processes associated with the embryo samples (Figure 6B, Table S3B). However, without *Blattabacterium* reads, the potential functional category, hydrocarbon degradation, was very prominent in the Ayayee_Embryo samples, but entirely negligible in the Jennings_Embryo samples (Figure 6B, Table S3B). In contrast, human gut, human pathogens, human‐associated, ureolysis, and mammalian gut microbial potential functional categories were abundant in the Jennings_Embryo samples. As with the analyses with Blattabacterial reads above, this may reflect the commensal nature of gut microbiota membership. However, contamination and nonspecific classification cannot be ruled out, ultimately highlighting the limitations of inferring potential functional categories from partial 16S amplicon data.

**FIGURE 6:**
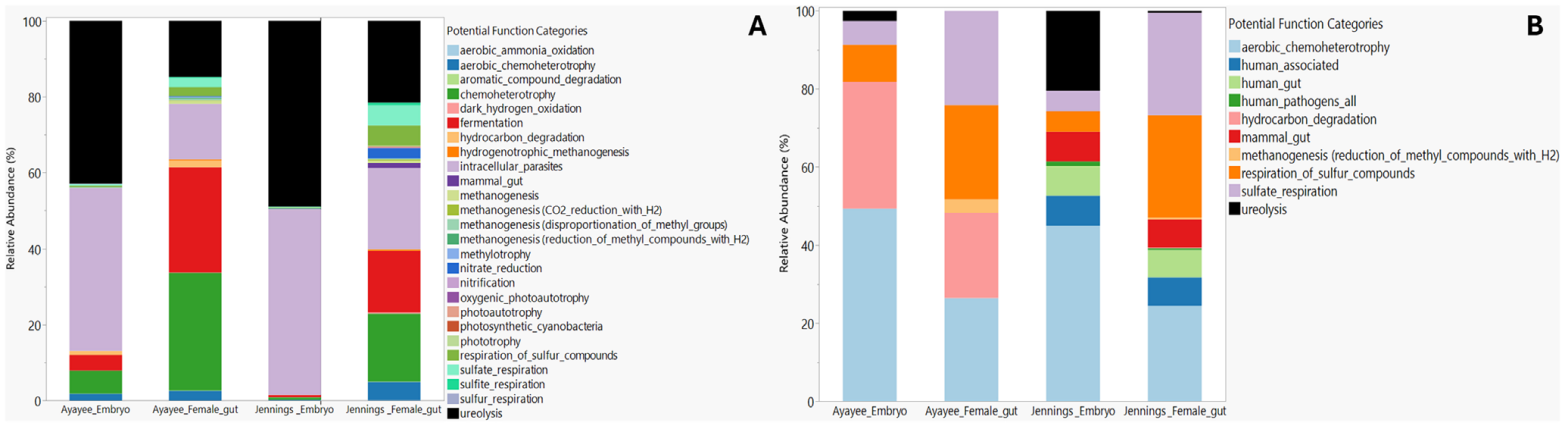
(A) The relative abundances of 26 differentially abundant potential bacterial metabolic functional groups highlighting distinct functional potentials among sample categories *with Blattabacterium reads* (*Kruskal–Wallis; FDR‐adjusted p‐value = 0.05*), and (B) the relative abundances of 10 differentially abundant potential bacterial metabolic functional groups highlighting distinct functional potentials among sample categories *without Blattabacterium reads* (*Kruskal–Wallis; FDR‐adjusted p‐value = 0.05*) after FAPROTAX analyses.

## 4 Discussion

Insect–bacterial associations can strongly influence host fitness and facilitate adaptive radiation (Janson et al. 2008; Vavre and Kremer 2014). However, gut microbial associates and bacteriocyte‐residing (obligate) endosymbionts should be distinguished when examining gut microbiome ecology, as their acquisition, dynamics, functions, and selection pressures differ (Gupta and Nair 2020). Accordingly, recent microbial ecology studies favor removing endosymbiont reads from 16S amplicon datasets when investigating ecological drivers of gut microbiome composition, as endosymbionts can influence the density, diversity, and abundance of non‐endosymbiotic bacteria within hosts (Basit et al. 2025; Guse and Pietri 2024; Simhadri et al. 2017; Li et al. 2022; Mazel et al. 2024). Here, we reexamined the only available datasets describing maternal and associated embryonic microbiomes of the viviparous cockroach *Diploptera punctata* (Ayayee et al. 2017; Jennings et al. 2019). We combined the datasets and analyzed them with and without *Blattabacterium* reads to characterize embryonic non‐*Blattabacterium* taxa and, using taxonomy‐based functional inference, generate hypotheses about the potential functional capabilities of these acquired bacteria.

### 4.1 Composition of Embryonic Non‐Blattabacterial Microbiomes

Despite extensive research on the composition, ecology, protein structure, and molecular regulation of nutritive milk secretions in gravid females (Banerjee et al. 2016; Evans and Stay 1995; Jennings et al. 2020; Williford et al. 2004), it remains unknown whether this secretion is sterile or serves as a route for non‐endosymbiotic microbial transmission, as reported in the tsetse fly, *Glossina morsitans* (Attardo et al. 2008).

In this study, after excluding *Blattabacterium* reads, substantial differences in diversity and community composition of the embryonic microbiomes were observed between the Jennings and Ayayee samples. Jennings_Embryo samples were dominated by only two non‐blattabacterial families (*Enterobacteriaceae* and D*ysgonomonadaceae*), whereas Ayayee_Embryo samples contained four dominant families (*Fusobacteriaceae, Halomonadaceae*, Unassigned Alteromonadales, and Unassigned Gammaproteobacteria). The detection of non‐blattabacterial taxa in both datasets suggests their potential acquisition by embryos, although differences in laboratory environment, rearing conditions, diet, and processing methods may contribute to these patterns. Importantly, these findings challenge the interpretation of Jennings et al. (2019) that non‐blattabacterial sequences were inconsequential to embryonic development or primarily reflected sequencing artifacts or taxonomic misidentifications. Collectively, our findings indicate that Jennings_Embryo samples possess a substantially reduced and less diverse microbiome relative to Ayayee_Embryo samples, consistent with the lower diversity and restricted composition observed in Jennings_Female_gut compared with Ayayee_Female_gut microbiomes. These corroborating data suggest possible context‐dependent acquisition of microbes by developing embryos.

The differences in non‐blattabacterial gut microbiome composition between the Ayayee_Female_gut and Jennings_Female_gut samples may reflect differences in environmental conditions (e.g., diet and husbandry) and methodological practices (e.g., surface sterilization, dissection, and sample handling) between studies. The greater maternal microbiome diversity observed in the Ayayee samples coincided with greater embryonic microbiome diversity, whereas both were lower in the Jennings samples, suggesting a potential environmental or laboratory‐dependent effect on these maternal and embryonic microbiomes. Differences in acclimation duration and diet may provide additional explanations for these patterns. Cockroaches used in the Jennings study were acclimated for 1 week after transfer from The Ohio State University Biological Sciences Greenhouse Insectary (Jennings et al. 2019). Although cockroaches can turn over gut contents within 7 days, studies assessing diet‐associated microbiome changes commonly allow ≥ 10 days following dietary shifts (Ayayee et al. 2024; Carrasco et al. 2015; Contreras and Lorenz 2016; Dockman and Ottesen 2024). Thus, the shorter acclimation period may have limited microbiome turnover or establishment of microbiota associated with the new diet. This possibility is consistent with the lower, though not statistically significant, diversity reported for OSU mothers compared with UC mothers by Jennings et al. (2019). Dietary history may have further contributed to differences in maternal microbiome composition. Cockroaches used in the Ayayee study originated from a UC colony historically maintained on Red Flannel Hi‐Protein Formula dog food (PMI Nutrition, St. Louis, MO, USA) (Ayayee et al. 2017), whereas the OSU Insectary colony used in Jennings et al. (2019) was maintained on Tetramin fish food (Spectrum Brands Pet). Collectively, differences in colony history, diet, acclimation, and experimental procedures may have contributed to the contrasting maternal and embryonic microbiome profiles observed between studies.

Overall, differences in microbiome diversity and composition between embryonic and maternal samples confirm the presence of non‐blattabacterial bacteria associated with developing *Diploptera* embryos. However, further research is needed to determine whether these taxa are stable embryo‐associated bacteria or transient environmental or contaminant signals. Their detection nonetheless provides a basis for exploring potential functions and developing hypotheses for future research.

### 4.2 Predicted Potential Functional Capabilities of Embryonic Non‐Blattabacterial Microbiomes

Despite the reduced diversity and compositional differences among embryonic non‐blattabacterial taxa, we identified a limited set of potential functional categories—not actual functions—that provide a starting point for hypotheses about their biological relevance. These hypotheses require experimental validation. First, the “milk” secreted by gravid females to developing embryos in the brood sac is deficient in tryptophan and methionine (Williford et al. 2004), and the *Blattabacterium* endosymbiont lacks pathways for their biosynthesis (Hojo et al. 2013; Kambhampati et al. 2013; Sabree et al. 2009). In a previous study, embryo‐associated *Halomonadaceae* and *Shewanellaceae* (two maternally associated and environmentally acquired gut bacterial residents) were therefore proposed as potential sources of these essential amino acids in *Diploptera* embryos (Ayayee et al. 2017), because both have complete methionine and tryptophan biosynthetic pathways (Jabri et al. 2012; Santos‐Garcia et al. 2014). More recently, gut bacteria in adult *Diploptera* females were shown to provide essential amino acids (Ayayee et al. 2024). Thus, the detection of *Halomonadaceae* and other sulfur‐reducing bacteria in embryonic microbiomes in this study provides an opportunity to test whether these non‐blattabacterial taxa similarly contribute to the provisioning of essential amino acids during embryonic development.

FAPROTAX analyses identified potential bacterial functional categories associated with sulfate reduction and sulfur respiration, suggesting that these taxa may possibly contribute to the biosynthesis of sulfur‐containing amino acids such as methionine and cysteine in *Diploptera* (Braccia et al. 2021; Kabil et al. 2014; Kim et al. 2024). Similarly, the prominence of hydrocarbon degradation functions in the Ayayee_Embryo samples may indicate microbial contributions to tryptophan‐related metabolism through the aryl hydrocarbon receptor (*AhR*) pathway (Ghiboub et al. 2020; Zelante et al. 2013) and polycyclic aromatic hydrocarbon (PAH) degradation pathways (Ghosal et al. 2016; Wang et al. 2018). The enrichment of ureolytic functions in non‐*Blattabacterium* embryonic microbiomes further suggests that these taxa may potentially contribute to urea hydrolysis and nitrogen provisioning, complementing the nitrogen contributions of the fat‐body‐residing endosymbiont *Blattabacterium* (Hojo et al. 2013; Murugan et al. 2021; Sabree et al. 2009; Shen et al. 2025). Together, these findings point to several potentially important metabolic functions, but they remain hypothetical and speculative and require experimental validation before any biological relevance can be attributed to non‐Blattabacterium taxa associated with developing embryos. Several approaches could help determine their functional significance. First, cockroach endosymbiont and gut microbial abundances could be experimentally reduced using rifampicin (Muñoz‐Benavent et al. 2021; Rosas et al. 2018) or chlortetracycline (Pablo et al. 2018) to generate quasi‐aposymbiotic third‐ or fourth‐generation colonies. In these colonies, 16S amplicon sequencing of maternal and embryonic microbiomes, excluding *Blattabacterium* reads, could help assess the extent of non‐Blattabacterium acquisition by quasi‐aposymbiotic embryos. RNA‐seq could then be used to assess differential gene expressions across associated *Blattabacterium* and non‐Blattabacterium associates, providing a first step toward linking embryonic microbiome composition with function. Additional insight could be gained by characterizing female “milk” secretions from treated and control colonies using X‐ray diffraction and mass spectrometry (Banerjee et al. 2016). Finally, the metabolic costs of experimentally reducing microbial associates could be evaluated by measuring compensatory changes in standard metabolic rate in aposymbiotic females and their progeny. Together, these approaches would help distinguish predicted metabolic potential from functions that are actively expressed and biologically relevant during embryo development.

## 5 Conclusion and Future Areas of Research

Overall, reexamination of the two available 16S amplicon datasets from *Diploptera punctata* mothers and embryos revealed several new insights. Non‐blattabacterial reads were detected in developing embryos. Associated maternal gut microbiomes showed possible environment‐ (laboratory) and diet‐specific differences. These differences in maternal microbiomes may influence the presence and composition of embryonic microbiomes, regardless of their source (maternal, environmental, or contaminant). 16S amplicon data limit functional interpretation; these findings highlight potential avenues for investigating the biological roles of non‐blattabacterial microbes associated with the developing embryos. Further research into maternal and embryonic microbiomes will help clarify the complex factors shaping microbial communities in this viviparous cockroach.

## Author Contributions

**Brennan Jonas:** data curation (equal), formal analysis (equal), investigation (equal), methodology (equal), validation (equal), visualization (equal), writing – review and editing (equal). **Victoria Arrowsmith:** data curation (equal), investigation (equal), methodology (equal), validation (equal), writing – review and editing (equal). **Paul Ayayee:** conceptualization (lead), data curation (supporting), formal analysis (equal), methodology (equal), project administration (equal), resources (lead), software (lead), supervision (lead), validation (equal), visualization (lead), writing – original draft (lead), writing – review and editing (lead).

## Funding

The project was funded with start‐up funds from the College of Arts and Sciences and the Biology Department at the University of Nebraska at Omaha, provided to Paul Ayayee. This work was also completed using the University of Nebraska DNA Sequencing Core, which receives partial support from the National Institute of General Medical Sciences (NIGMS) INBRE‐P20GM103427‐19 grant and the Fred & Pamela Buffett Cancer Center Support Grant‐P30 CA036727.

## Ethics Statement

No animal rights were violated in the execution of this study. UNO's Institutional Animal Care and Use 278 Committee (IACUC) did not apply to the use of insects.

## Conflicts of Interest

The authors declare no conflicts of interest.

## Supporting information


**Table S1:** Summary of quality preprocessing steps and reads retained across analyzed samples from both publications.
**Table S2:** (A) Abundances of 55 bacterial families that were significantly different (*p < 0.05*) among the sample categories analyzed with *Blattabacteriaceae* reads retained in the analyses, and (B) the 16 differentially abundant bacterial families (at *p* < 0.05) among the sample categories analyzed without *Blattabacteriaceae* reads.
**Table S3:** (A) Abundances of 26 potential bacterial functional pathways significantly different (*p < 0.05*) among the sample categories analyzed *with Blattabacteriaceae reads* retained in the analyses, and (B) the 10 differentially abundant potential bacterial functional pathways (*at p < 0.05*) among the sample categories analyzed *without Blattabacteriaceae reads*.

## Data Availability

The data used in this study are openly available in the NCBI Sequence Read Archive (SRA) database under the accession numbers PRJNA522760 for the Jennings et al. 2019 dataset and PRJNA314839 for the Ayayee et al. 2017 dataset. All analyses performed with these data are present in the manuscript and the supporting information associated with this submission.
