# Supplementary material for: Does Mother Provide? Re‐Examination of Maternal and Embryonic Microbiomes of Diploptera punctata
Source: Ecol Evol. 2026 Oct 12;16(10):e74504. doi: 10.1002/ece3.74504 (PMC13657858; doi:10.1002/ece3.74504)
Supplement: Supplementary file 1 [file ECE3-16-e74504-s001.docx]

**Table S1.** Summary of quality preprocessing steps and reads retained across analyzed samples from both publications.

| **Data Source** | **Sample** | **Raw Reads** | **Filtered 150bp/2e** | **Merged pairs** | **Non-chimeric reads** | **Data retained** |
| --- | --- | --- | --- | --- | --- | --- |
| (Ayayee 2017)_female | SRR3216406 | 139961 | 104204 | 102014 | 53966 | 38.6% |
| (Ayayee 2017)_embryo | SRR3216408 | 158218 | 82388 | 81746 | 48759 | 30.8% |
| (Ayayee 2017)_embryo | SRR3216410 | 100998 | 88817 | 88433 | 37774 | 37.4% |
| (Ayayee 2017)_female_gut | SRR3216412 | 61764 | 48668 | 47899 | 19996 | 32.4% |
| (Ayayee 2017)_embryo | SRR3216414 | 122451 | 98348 | 95480 | 42669 | 34.8% |
| (Ayayee 2017)_female_gut | SRR3216416 | 118121 | 103154 | 100987 | 42484 | 36.0% |
| (Ayayee 2017)_embryo | SRR3216418 | 122070 | 74477 | 73124 | 44561 | 36.5% |
| (Ayayee 2017)_female_gut | SRR3216419 | 107181 | 70801 | 69041 | 31887 | 29.8% |
| (Ayayee 2017)_embryo | SRR3216421 | 68588 | 57692 | 56532 | 32100 | 46.8% |
| (Ayayee 2017)_embryo | SRR3216423 | 189989 | 162416 | 161971 | 93298 | 49.1% |
| (Ayayee 2017)_female_gut | SRR3216425 | 117725 | 103707 | 101003 | 38991 | 33.1% |
| (Ayayee 2017)_embryo | SRR3216427 | 127029 | 99464 | 98528 | 49805 | 39.2% |
| (Ayayee 2017)_female_gut | SRR3216430 | 98639 | 87052 | 84306 | 38602 | 39.1% |
| (Ayayee 2017)_embryo | SRR3216432 | 113913 | 93139 | 92167 | 44601 | 39.2% |
| (Ayayee 2017)_female_gut | SRR3216434 | 192957 | 59611 | 58396 | 22363 | 11.6% |
| (Jennings 2019)_female_gut | SRR8588775 | 18630 | 18233 | 17449 | 16809 | 90.2% |
| (Jennings 2019)_embryo | SRR8588776 | 33603 | 32964 | 32128 | 31090 | 92.5% |
| (Jennings 2019)_female_gut | SRR8588777 | 89839 | 87184 | 86114 | 84917 | 94.5% |
| (Jennings 2019)_embryo | SRR8588778 | 98163 | 96334 | 94576 | 93808 | 95.6% |
| (Jennings 2019)_embryo | SRR8588779 | 3995 | 3883 | 3791 | 3781 | 94.6% |
| (Jennings 2019)_embryo | SRR8588780 | 9735 | 9504 | 9425 | 9425 | 96.8% |
| (Jennings 2019)_embryo | SRR8588781 | 7056 | 6955 | 6904 | 6904 | 97.8% |
| (Jennings 2019)_embryo | SRR8588782 | 13885 | 13616 | 13536 | 13536 | 97.5% |
| (Jennings 2019)_embryo | SRR8588783 | 5077 | 4945 | 4886 | 4886 | 96.2% |
| (Jennings 2019)_embryo | SRR8588784 | 51739 | 50640 | 50376 | 50202 | 97.0% |
| (Jennings 2019)_embryo | SRR8588785 | 93057 | 91187 | 89707 | 89106 | 95.8% |
| (Jennings 2019)_embryo | SRR8588794 | 128481 | 125914 | 124432 | 123063 | 95.8% |
| (Jennings 2019)_embryo | SRR8588803 | 43725 | 42661 | 42124 | 41982 | 96.0% |
| (Jennings 2019)_embryo | SRR8588804 | 109810 | 88203 | 87142 | 84950 | 77.4% |
| (Jennings 2019)_embryo | SRR8588808 | 29736 | 29130 | 28249 | 27392 | 92.1% |
| (Jennings 2019)_embryo | SRR8588809 | 39624 | 38544 | 37925 | 37122 | 93.7% |
| (Jennings 2019)_embryo | SRR8588810 | 110210 | 107180 | 105608 | 103443 | 93.9% |
| (Jennings 2019)_embryo | SRR8588813 | 115239 | 92155 | 90472 | 87523 | 75.9% |
| (Jennings 2019)_embryo | SRR8588814 | 31823 | 22315 | 21642 | 20827 | 65.4% |
| (Jennings 2019)_female_gut | SRR8588828 | 32141 | 28687 | 27968 | 27311 | 85.0% |
| (Jennings 2019)_female_gut | SRR8588829 | 58018 | 47542 | 47406 | 47404 | 81.7% |
| (Jennings 2019)_female_gut | SRR8588830 | 40460 | 31403 | 31229 | 31220 | 77.2% |
| (Jennings 2019)_female_gut | SRR8588831 | 46214 | 40676 | 40608 | 40605 | 87.9% |
| (Jennings 2019)_female_gut | SRR8588832 | 57480 | 50919 | 50629 | 50629 | 88.1% |
| (Jennings 2019)_female_gut | SRR8588833 | 44799 | 34491 | 34333 | 34326 | 76.6% |
| (Jennings 2019)_female_gut | SRR8588761 | 21482 | 15540 | 15389 | 15386 | 71.6% |
| (Jennings 2019)_female_gut | SRR8588762 | 34727 | 27292 | 27167 | 27165 | 78.2% |
| (Jennings 2019)_female_gut | SRR8588786 | 44804 | 36130 | 35999 | 35990 | 80.3% |
| (Jennings 2019)_female_gut | SRR8588787 | 31301 | 22694 | 22470 | 22455 | 71.7% |
| (Jennings 2019)_female_gut | SRR8588788 | 26870 | 20459 | 20324 | 20321 | 75.6% |
| (Jennings 2019)_female_gut | SRR8588789 | 61138 | 48621 | 47800 | 46507 | 76.1% |
| (Jennings 2019)_female_gut | SRR8588792 | 90559 | 72119 | 70942 | 68782 | 76.0% |
| (Jennings 2019)_female_gut | SRR8588793 | 31576 | 27080 | 26369 | 25951 | 82.2% |
| (Jennings 2019)_embryo | SRR8588805 | 47644 | 42480 | 42370 | 42370 | 88.9% |
| (Jennings 2019)_embryo | SRR8588806 | 89019 | 72728 | 71527 | 69334 | 77.9% |
| (Jennings 2019)_embryo | SRR8588807 | 21903 | 16595 | 15878 | 15356 | 70.1% |
| (Jennings 2019)_female_gut | SRR8588820 | 23692 | 23139 | 22697 | 22422 | 94.6% |
| (Jennings 2019)_female_gut | SRR8588821 | 43530 | 37775 | 37225 | 36890 | 84.7% |
| (Jennings 2019)_female_gut | SRR8588822 | 106694 | 85102 | 83918 | 81276 | 76.2% |
| (Jennings 2019)_embryo | SRR8588823 | 59972 | 57796 | 56966 | 55623 | 92.7% |
| (Jennings 2019)_female_gut | SRR8588825 | 96408 | 76580 | 75174 | 72361 | 75.1% |
| (Jennings 2019)_embryo | SRR8588838 | 47239 | 35959 | 35763 | 35747 | 75.7% |
| **Sum** |  | **4030701** | **3247292** | **3198264** | **2498053** | **41.17314933** |

**Table S2: A).** Abundances of 55 bacterial families that were significantly different (*P < 0.05*) among the sample categories analyzed **with *Blattabacteriaceae* reads** retained in the analyses, and **B)** the 16 differentially abundant bacterial families (at P < 0.05) among the sample categories **analyzed without** *Blattabacteriaceae* reads.

| **A. Differentially abundant Families with Blattabacterium** | **Ayayee_Embryo** | **Ayayee_Female_gut** | **Jennings _Embryo** | **Jennings _Female_gut** |
| --- | --- | --- | --- | --- |
| *Blattabacteriaceae* | 521.25 | 90.86 | 0.00 | 0.00 |
| *Blattabacteriaceae* | 248.50 | 50.29 | 0.00 | 0.00 |
| *Blattabacteriaceae* | 240.63 | 48.00 | 0.00 | 0.00 |
| *Blattabacteriaceae* | 223.75 | 43.14 | 0.00 | 0.00 |
| *Blattabacteriaceae* | 179.75 | 38.00 | 0.00 | 0.00 |
| *Blattabacteriaceae* | 138.00 | 25.14 | 0.00 | 0.00 |
| *Blattabacteriaceae* | 123.88 | 20.14 | 0.00 | 0.00 |
| *Blattabacteriaceae* | 220.75 | 41.14 | 0.00 | 0.00 |
| *Blattabacteriaceae* | 192.13 | 35.43 | 0.00 | 0.00 |
| *Blattabacteriaceae* | 168.13 | 32.00 | 0.00 | 0.00 |
| *Unassigned Alteromonadales* | 6.63 | 0.86 | 0.00 | 0.00 |
| *Unmassigned Gammaproteobacteria* | 14.75 | 1.00 | 0.00 | 0.00 |
| *Blattabacteriaceae* | 0.00 | 0.00 | 3483.59 | 956.10 |
| *Desulfobacteraceae* | 0.00 | 0.00 | 0.27 | 13.45 |
| *Tannerellaceae* | 0.00 | 0.00 | 0.09 | 17.05 |
| *Halomonadaceae* | 15.38 | 3.43 | 0.00 | 0.00 |
| *Halomonadaceae* | 26.63 | 6.43 | 0.00 | 0.00 |
| *Unassigned Bacteroidia* | 0.00 | 5.57 | 0.00 | 0.00 |
| *Tannerellaceae* | 0.00 | 3.71 | 0.00 | 0.00 |
| *Unassigned Bacteroidia* | 0.00 | 2.14 | 0.00 | 0.00 |
| *Enterococcaceae* | 0.00 | 7.86 | 0.00 | 0.00 |
| *Rikenellaceae* | 0.00 | 3.43 | 0.00 | 0.00 |
| *Unassigned Bacteroidia* | 0.00 | 2.00 | 0.00 | 0.00 |
| *Fusobacteriaceae* | 0.00 | 23.57 | 0.00 | 0.00 |
| *Fusobacteriaceae* | 0.00 | 18.71 | 0.00 | 0.00 |
| *Unassigned Bacteroidales* | 0.00 | 12.86 | 0.00 | 0.00 |
| *Desulfobacteraceae* | 0.00 | 8.14 | 0.00 | 0.00 |
| *Unassigned Bacteroidia* | 0.00 | 9.43 | 0.00 | 0.00 |
| *vadinHA21* | 0.00 | 7.43 | 0.00 | 0.00 |
| *Desulfobacteraceae* | 0.00 | 8.86 | 0.00 | 0.00 |
| *Rikenellaceae* | 0.00 | 7.43 | 0.00 | 0.00 |
| *Enterobacteriaceae* | 0.00 | 6.57 | 0.00 | 0.00 |
| *Ruminococcaceae* | 0.00 | 5.14 | 0.00 | 0.00 |
| *Rikenellaceae* | 0.00 | 3.71 | 0.00 | 0.00 |
| *Fusobacteriaceae* | 0.00 | 24.86 | 0.00 | 0.00 |
| *Fusobacteriaceae* | 0.00 | 27.00 | 0.00 | 0.00 |
| *Fusobacteriaceae* | 0.00 | 15.86 | 0.00 | 0.00 |
| *Tannerellaceae* | 0.00 | 9.71 | 0.00 | 0.00 |
| *Rikenellaceae* | 0.00 | 11.14 | 0.00 | 0.00 |
| *Unassigned Bacteroidia* | 0.00 | 12.14 | 0.00 | 0.00 |
| *Methanosarcinaceae* | 0.00 | 5.43 | 0.00 | 0.00 |
| *Ruminococcaceae* | 0.00 | 0.00 | 0.14 | 5.60 |
| *Lachnospiraceae* | 0.00 | 0.00 | 0.09 | 13.90 |
| *Desulfovibrionaceae* | 0.00 | 0.00 | 0.23 | 22.90 |
| *Acetobacteraceae* | 0.00 | 0.00 | 0.23 | 11.85 |
| *Synergistaceae* | 0.00 | 0.00 | 0.18 | 6.30 |
| *Enterobacteriaceae* | 0.00 | 0.00 | 3.77 | 108.95 |
| *Dysgonomonadaceae* | 0.00 | 0.00 | 1.05 | 13.25 |
| *Dysgonomonadaceae* | 0.00 | 0.00 | 0.05 | 13.05 |
| *Unassigned Rs-K70_termite_group* | 0.00 | 0.00 | 0.77 | 29.75 |
| *Desulfovibrionaceae* | 0.00 | 0.00 | 0.18 | 9.70 |
| *Unassigned Clostridiales* | 0.00 | 0.00 | 0.27 | 11.10 |
| *Unassigned Clostridiales* | 0.00 | 0.00 | 0.00 | 5.90 |
| *Ruminococcaceae* | 0.00 | 0.00 | 0.00 | 3.85 |
| *Tannerellaceae* | 0.00 | 0.00 | 0.00 | 3.95 |
| *Desulfovibrionaceae* | 0.00 | 0.00 | 0.00 | 10.70 |
| *Synergistaceae* | 0.00 | 0.00 | 0.00 | 7.75 |
| *Ruminococcaceae* | 0.00 | 0.00 | 0.00 | 10.05 |
| *Ruminococcaceae* | 0.00 | 0.00 | 0.00 | 5.95 |
| *Dysgonomonadaceae* | 0.00 | 0.00 | 2.09 | 53.80 |
| *Dysgonomonadaceae* | 0.00 | 0.00 | 0.59 | 15.35 |
| *Enterobacteriaceae* | 0.00 | 0.00 | 0.59 | 85.45 |
| *Fusobacteriaceae* | 0.38 | 24.14 | 0.00 | 0.00 |
| *Dysgonomonadaceae* | 0.13 | 18.71 | 0.00 | 0.00 |
| *Fusobacteriaceae* | 0.13 | 24.43 | 0.00 | 0.00 |
| *Paludibacteraceae* | 0.13 | 10.71 | 0.00 | 0.00 |
| *PeH15* | 0.00 | 0.00 | 2.77 | 48.80 |
| *Unassigned LD1-PB3* | 0.00 | 0.00 | 1.05 | 11.35 |
| *Tannerellaceae* | 0.00 | 0.00 | 0.14 | 9.75 |
| *Rikenellaceae* | 0.00 | 0.00 | 1.09 | 11.50 |
| *Unassigned Clostridiales* | 0.00 | 0.00 | 0.09 | 4.70 |
| *Staphylococcaceae* | 0.00 | 0.00 | 0.09 | 3.65 |
| *Bacteroidaceae* | 0.00 | 0.00 | 0.00 | 1.90 |
| *Coriobacteriales_Incertae_Sedis* | 0.00 | 0.00 | 0.00 | 2.90 |
| *Lachnospiraceae* | 0.00 | 0.00 | 0.00 | 5.85 |
| *Unassigned Rhizobiales* | 0.00 | 0.00 | 0.00 | 4.35 |
| *Ruminococcaceae* | 0.00 | 0.00 | 0.00 | 4.95 |
| *Synergistaceae* | 0.00 | 0.00 | 0.00 | 12.70 |
| *Dysgonomonadaceae* | 0.00 | 0.00 | 0.00 | 7.85 |
| *Fusobacteriaceae* | 0.75 | 17.43 | 0.00 | 0.00 |
| *Fusobacteriaceae* | 1.25 | 57.86 | 0.00 | 0.00 |
| *vadinHA21* | 0.38 | 17.14 | 0.00 | 0.00 |
| *Desulfobacteraceae* | 0.38 | 14.86 | 0.00 | 0.00 |
| *Rikenellaceae* | 0.00 | 0.00 | 0.23 | 6.10 |
| *Unassignd Bacteroidia* | 0.00 | 1.86 | 0.00 | 0.00 |
| *Methanosarcinaceae* | 0.00 | 1.57 | 0.00 | 0.00 |
| *Rikenellaceae* | 0.00 | 6.71 | 0.00 | 0.00 |
| *Dysgonomonadaceae* | 0.00 | 6.43 | 0.00 | 0.00 |
| *Tannerellaceae* | 0.00 | 7.57 | 0.00 | 0.00 |
| *Unassigned Rs-K70_termite_group* | 0.00 | 8.43 | 0.00 | 0.00 |
| *Methanosarcinaceae* | 0.00 | 4.29 | 0.00 | 0.00 |
| *Tannerellaceae* | 0.00 | 4.00 | 0.00 | 0.00 |
| *Tannerellaceae* | 0.00 | 5.71 | 0.00 | 0.00 |
| *Dysgonomonadaceae* | 0.00 | 4.57 | 0.00 | 0.00 |
| *Tannerellaceae* | 0.00 | 3.71 | 0.00 | 0.00 |
| *Tannerellaceae* | 0.00 | 3.57 | 0.00 | 0.00 |
| *Tannerellaceae* | 0.00 | 2.86 | 0.00 | 0.00 |
| *Ruminococcaceae* | 0.00 | 2.29 | 0.00 | 0.00 |
| *Rikenellaceae* | 0.00 | 1.86 | 0.00 | 0.00 |
| *Enterobacteriaceae* | 0.00 | 13.14 | 0.00 | 0.00 |
| *Enterobacteriaceae* | 0.00 | 8.57 | 0.00 | 0.00 |
| *Dysgonomonadaceae* | 0.00 | 6.86 | 0.00 | 0.00 |
| *Rikenellaceae* | 0.00 | 7.14 | 0.00 | 0.00 |
| *Tannerellaceae* | 0.00 | 7.29 | 0.00 | 0.00 |
| *Lachnospiraceae* | 0.00 | 7.29 | 0.00 | 0.00 |
| *Enterobacteriaceae* | 0.00 | 5.43 | 0.00 | 0.00 |
| *Rikenellaceae* | 0.00 | 5.43 | 0.00 | 0.00 |
| *Pseudomonadaceae* | 0.00 | 4.43 | 0.00 | 0.00 |
| *Rikenellaceae* | 0.00 | 4.71 | 0.00 | 0.00 |
| *Unassigned Bacteroidia* | 0.00 | 3.57 | 0.00 | 0.00 |
| *Unassigned Bacteroidia* | 0.00 | 4.43 | 0.00 | 0.00 |
| *Unassigned Bacteroidia* | 0.00 | 2.43 | 0.00 | 0.00 |
| *Dysgonomonadaceae* | 0.00 | 0.00 | 0.95 | 25.45 |
| *Ruminococcaceae* | 0.00 | 0.00 | 0.18 | 4.15 |
| *Desulfovibrionaceae* | 0.00 | 0.00 | 0.45 | 13.35 |
| *Enterococcaceae* | 0.00 | 0.00 | 1.59 | 19.55 |
| *Rikenellaceae* | 0.00 | 0.00 | 0.55 | 10.00 |
| *Desulfobacteraceae* | 0.00 | 0.00 | 5.09 | 41.85 |
| *Rikenellaceae* | 0.00 | 0.00 | 0.73 | 8.05 |
| *Ruminococcaceae* | 0.00 | 0.00 | 0.23 | 8.75 |
| *Ruminococcaceae* | 0.00 | 0.00 | 0.73 | 6.50 |
| *Corynebacteriaceae* | 0.00 | 0.00 | 0.23 | 6.85 |
| *Ruminococcaceae* | 0.00 | 0.00 | 0.77 | 4.20 |
| *Synergistaceae* | 0.00 | 0.00 | 0.14 | 17.55 |
| *Paludibacteraceae* | 0.00 | 0.00 | 0.00 | 2.50 |
| *Lachnospiraceae* | 0.00 | 0.00 | 0.00 | 4.85 |
| *Christensenellaceae* | 0.00 | 0.00 | 0.00 | 3.95 |
| *Dysgonomonadaceae* | 0.00 | 0.00 | 0.00 | 9.25 |
| *Unassigned vadinHA49* | 0.00 | 0.00 | 0.27 | 7.90 |
| *Unassigned Corynebacteriales* | 0.00 | 0.00 | 0.14 | 3.15 |
| *Ruminococcaceae* | 0.00 | 0.00 | 0.18 | 6.45 |
| *Unassignd Clostridiales* | 0.00 | 0.00 | 0.18 | 3.30 |
| *Fusobacteriaceae* | 0.00 | 0.00 | 0.27 | 54.90 |
| *Unassigned Bacteroidia* | 1.38 | 2.86 | 0.00 | 0.00 |
| *Erysipelotrichaceae* | 0.00 | 0.00 | 0.18 | 2.90 |
| *Ruminococcaceae* | 0.00 | 0.00 | 1.00 | 12.40 |
| *Enterococcaceae* | 0.00 | 0.00 | 0.05 | 15.90 |
| *Ruminococcaceae* | 0.00 | 0.00 | 0.05 | 3.15 |
| *Ruminococcaceae* | 0.00 | 0.00 | 0.05 | 4.50 |
| *Unassigned Alteromonadales* | 2.88 | 1.71 | 0.00 | 0.00 |
| *Tannerellaceae* | 0.00 | 0.00 | 0.09 | 4.30 |
| *Bacteroidaceae* | 0.25 | 4.57 | 0.00 | 0.00 |
| *Tannerellaceae* | 0.13 | 6.57 | 0.00 | 0.00 |
| *Enterobacteriaceae* | 0.63 | 8.71 | 0.00 | 0.00 |
| *Rikenellaceae* | 0.00 | 0.00 | 0.00 | 3.75 |
| *Bacteroidaceae* | 0.00 | 0.00 | 0.00 | 2.85 |
| *Christensenellaceae* | 0.00 | 0.00 | 0.00 | 2.10 |
| *Ruminococcaceae* | 0.00 | 0.00 | 0.00 | 2.20 |
| *Dysgonomonadaceae* | 0.00 | 0.00 | 0.00 | 5.75 |
| *Coriobacteriales_Incertae_Sedis* | 0.00 | 0.00 | 0.00 | 2.30 |
| *Desulfobacteraceae* | 0.00 | 0.00 | 0.00 | 13.35 |
| *Desulfovibrionaceae* | 0.00 | 0.00 | 0.00 | 10.85 |
| *Rikenellaceae* | 0.00 | 0.00 | 0.14 | 4.15 |
| *Unassigned Clostridiales* | 0.00 | 0.00 | 0.14 | 4.00 |
| *Unassigned vadinHA49* | 0.25 | 3.57 | 0.00 | 0.00 |
| *Rikenellaceae* | 0.00 | 0.00 | 0.27 | 9.20 |
| *Unassigned Saccharimonadales* | 0.00 | 0.00 | 0.09 | 4.50 |
| *Family_XIII* | 0.00 | 0.00 | 0.18 | 3.25 |
| *Synergistaceae* | 0.00 | 0.00 | 0.23 | 4.55 |
| *Synergistaceae* | 0.00 | 0.00 | 0.27 | 18.30 |
| *Synergistaceae* | 0.00 | 0.00 | 0.50 | 7.00 |
| *Unassigned LD1-PB3* | 0.00 | 0.00 | 0.27 | 2.90 |
| *Rikenellaceae* | 0.00 | 0.00 | 0.64 | 7.35 |
| *Ruminococcaceae* | 0.00 | 0.00 | 0.36 | 4.25 |
| *Unassigned Rs-K70_termite_group* | 0.00 | 0.00 | 0.09 | 9.50 |
| *Ruminococcaceae* | 0.00 | 0.00 | 0.45 | 4.15 |
| *Ruminococcaceae* | 0.00 | 0.00 | 1.00 | 9.55 |
| *Syntrophomonadaceae* | 0.00 | 0.00 | 0.32 | 1.70 |
| *Unassigned WCHB1-41* | 0.00 | 0.00 | 1.14 | 4.90 |
| *Ruminococcaceae* | 0.00 | 1.43 | 0.00 | 0.00 |
| *Rikenellaceae* | 0.00 | 0.86 | 0.00 | 0.00 |
| *Enterobacteriaceae* | 0.00 | 15.29 | 0.00 | 0.00 |
| *Rikenellaceae* | 0.00 | 10.86 | 0.00 | 0.00 |
| *Unassigned Bacteroidia* | 0.00 | 6.86 | 0.00 | 0.00 |
| *Unassigned Bacteroidia* | 0.00 | 4.71 | 0.00 | 0.00 |
| *Unassigned Betaproteobacteriales* | 0.00 | 6.14 | 0.00 | 0.00 |
| *Enterococcaceae* | 0.00 | 5.29 | 0.00 | 0.00 |
| *Tannerellaceae* | 0.00 | 2.57 | 0.00 | 0.00 |
| *Desulfovibrionaceae* | 0.00 | 1.86 | 0.00 | 0.00 |
| *Ruminococcaceae* | 0.00 | 1.86 | 0.00 | 0.00 |
| *Rikenellaceae* | 0.00 | 2.00 | 0.00 | 0.00 |
| *Unassigned vadinHA49* | 0.00 | 1.14 | 0.00 | 0.00 |
| *Tannerellaceae* | 0.00 | 1.43 | 0.00 | 0.00 |
| *Enterococcaceae* | 0.00 | 1.57 | 0.00 | 0.00 |
| *Unassigned Bacteroidia* | 0.00 | 1.29 | 0.00 | 0.00 |
| *Enterococcaceae* | 0.00 | 1.14 | 0.00 | 0.00 |
| *Streptococcaceae* | 0.00 | 7.86 | 0.00 | 0.00 |
| *Enterobacteriaceae* | 0.00 | 6.86 | 0.00 | 0.00 |
| *Paludibacteraceae* | 0.00 | 4.57 | 0.00 | 0.00 |
| *Ruminococcaceae* | 0.00 | 3.86 | 0.00 | 0.00 |
| *Tannerellaceae* | 0.00 | 4.29 | 0.00 | 0.00 |
| *Rikenellaceae* | 0.00 | 3.71 | 0.00 | 0.00 |
| *Dysgonomonadaceae* | 0.00 | 4.14 | 0.00 | 0.00 |
| *Unassigned Proteobacteria* | 0.00 | 3.43 | 0.00 | 0.00 |
| *Unassigned Bacteroidales* | 0.00 | 2.86 | 0.00 | 0.00 |
| *Dysgonomonadaceae* | 0.00 | 3.57 | 0.00 | 0.00 |
| *Methanomethylophilaceae* | 0.00 | 3.14 | 0.00 | 0.00 |
| *Ruminococcaceae* | 0.00 | 2.00 | 0.00 | 0.00 |
| *Desulfovibrionaceae* | 0.00 | 2.29 | 0.00 | 0.00 |
| *Unassigned Clostridiales* | 0.00 | 0.00 | 0.09 | 3.40 |
| *Family_XIII* | 0.00 | 0.00 | 0.00 | 1.90 |
| *Coriobacteriales_Incertae_Sedis* | 0.00 | 0.00 | 0.00 | 1.50 |
| *Pirellulaceae* | 0.00 | 0.00 | 0.00 | 2.00 |
| *Christensenellaceae* | 0.00 | 0.00 | 0.00 | 2.80 |
| *Peptococcaceae* | 0.00 | 0.00 | 0.00 | 2.25 |
| *Desulfovibrionaceae* | 0.00 | 0.00 | 0.00 | 2.10 |
| *Tannerellaceae* | 0.00 | 0.00 | 0.00 | 2.35 |
| *Christensenellaceae* | 0.00 | 0.00 | 0.00 | 2.65 |
| *Ruminococcaceae* | 0.00 | 0.00 | 0.00 | 3.30 |
| *Ruminococcaceae* | 0.00 | 0.00 | 0.00 | 4.10 |
| *Unassigned WCHB1-41* | 0.00 | 0.00 | 0.00 | 4.95 |
| *Pseudomonadaceae* | 0.00 | 0.00 | 0.00 | 5.75 |
| *Desulfovibrionaceae* | 0.00 | 0.00 | 0.00 | 4.60 |
| *Ruminococcaceae* | 0.00 | 0.00 | 0.09 | 2.60 |
| *Desulfovibrionaceae* | 0.00 | 0.00 | 0.23 | 9.10 |
| *M2PB4-65_termite_group* | 0.00 | 0.00 | 0.18 | 5.90 |
| *Ruminococcaceae* | 0.00 | 0.00 | 0.18 | 2.40 |
| *Rikenellaceae* | 0.00 | 0.00 | 0.18 | 2.40 |
| *Christensenellaceae* | 0.00 | 0.00 | 0.32 | 5.45 |
| *Unassigned Clostridiales* | 0.00 | 0.00 | 0.32 | 3.15 |
| *Peptostreptococcaceae* | 0.00 | 0.00 | 0.41 | 2.50 |
| *Paludibacteraceae* | 0.00 | 0.00 | 0.36 | 2.90 |
| *Desulfovibrionaceae* | 0.00 | 0.00 | 0.55 | 3.50 |
| *Ruminococcaceae* | 0.00 | 0.00 | 0.05 | 1.55 |
| *Unassigned Clostridiales* | 0.00 | 0.00 | 0.00 | 1.50 |
| *Ruminococcaceae* | 0.00 | 0.00 | 0.00 | 1.50 |
| *Rikenellaceae* | 0.00 | 0.00 | 0.00 | 2.00 |
| *Desulfovibrionaceae* | 0.00 | 0.00 | 0.00 | 1.75 |
| *Synergistaceae* | 0.00 | 0.00 | 0.00 | 2.20 |
| *Ruminococcaceae* | 0.00 | 0.00 | 0.00 | 2.65 |
| *Unassigned Clostridiales* | 0.00 | 0.00 | 0.00 | 3.10 |
| *Ruminococcaceae* | 0.00 | 0.00 | 0.00 | 2.40 |
| *M2PB4-65_termite_group* | 0.00 | 0.00 | 0.00 | 2.65 |
| *Dysgonomonadaceae* | 0.00 | 0.00 | 0.00 | 2.40 |
| *Dysgonomonadaceae* | 0.00 | 0.00 | 0.00 | 7.75 |
| *Ruminococcaceae* | 0.00 | 0.00 | 0.00 | 5.45 |
| *Lachnospiraceae* | 0.00 | 0.00 | 0.00 | 6.65 |
| *Dysgonomonadaceae* | 0.00 | 0.00 | 0.00 | 6.85 |
| *Unassigned Saccharimonadales* | 0.00 | 0.00 | 0.09 | 4.45 |
| *Lachnospiraceae* | 0.00 | 0.00 | 0.14 | 4.85 |
| **B. Differentially abundant Families with Blattbacterium** | **Ayayee_Embryo** | **Ayayee_Female_gut** | **Jennings _Embryo** | **Jennings _Female_gut** |
| *Unassigned Gammaproteobacteria* | 1.75 | 0.00 | 0.00 | 0.00 |
| *Halomonadaceae* | 6.88 | 0.29 | 0.00 | 0.00 |
| *Halomonadaceae* | 3.38 | 0.14 | 0.00 | 0.00 |
| *Enterobacteriaceae* | 0.00 | 0.00 | 0.18 | 5.60 |
| *Unassigned Rs-K70_termite_group* | 0.00 | 0.00 | 0.00 | 1.85 |
| *Unassigned Alteromonadales* | 1.50 | 0.00 | 0.00 | 0.00 |
| *Dysgonomonadaceae* | 0.00 | 0.00 | 0.55 | 2.85 |
| *Fusobacteriaceae* | 0.38 | 2.43 | 0.00 | 0.00 |
| *Tannerellaceae* | 0.00 | 0.00 | 0.00 | 1.05 |
| *Fusobacteriaceae* | 0.00 | 0.57 | 0.00 | 0.00 |
| *Unassigned Bacteroidales* | 0.00 | 0.57 | 0.00 | 0.00 |
| *Desulfobacteraceae* | 0.00 | 0.57 | 0.00 | 0.00 |
| *Paludibacteraceae* | 0.00 | 0.71 | 0.00 | 0.00 |
| *Tannerellaceae* | 0.00 | 0.71 | 0.00 | 0.00 |
| *Enterobacteriaceae* | 0.00 | 0.86 | 0.00 | 0.00 |
| *Fusobacteriaceae* | 0.00 | 1.43 | 0.00 | 0.00 |

**Table S3**. **A**). Abundances of 26 potential bacterial functional pathways significantly different (*P < 0.05*) among the sample categories analyzed ***with Blattabacteriaceae reads*** retained in the analyses, and **B**) the 10 differentially abundant potential bacterial functional pathways (*at P < 0.05*) among the sample categories analyzed ***without Blattabacteriaceae reads***.

| **A. Potential Microbial Functional Categories with *Blattabacteriaceae*** | **Ayayee_Embryo** | **Ayayee_Female_gut** | **Jennings _Embryo** | **Jennings _Female_gut** |
| --- | --- | --- | --- | --- |
| hydrocarbon_degradation | 66.63 | 75.43 | 0.00 | 1.45 |
| ureolysis | 2609.63 | 638.00 | 3596.77 | 976.05 |
| intracellular_parasites | 2605.88 | 635.14 | 3595.55 | 970.20 |
| sulfite_respiration | 1.50 | 13.57 | 1.82 | 31.90 |
| sulfate_respiration | 21.25 | 103.29 | 15.27 | 240.95 |
| chemoheterotrophy | 367.88 | 1340.43 | 46.05 | 811.80 |
| fermentation | 249.63 | 1200.00 | 41.00 | 736.40 |
| respiration_of_sulfur_compounds | 27.50 | 103.29 | 15.27 | 240.95 |
| dark_hydrogen_oxidation | 0.13 | 4.86 | 2.36 | 14.65 |
| methanogenesis | 4.50 | 34.29 | 3.73 | 26.25 |
| hydrogenotrophic_methanogenesis | 1.13 | 12.43 | 2.50 | 15.30 |
| methanogenesis (reduction_of_methyl_compounds_with_H2) | 0.00 | 9.43 | 0.14 | 0.75 |
| methanogenesis (CO2_reduction_with_H2) | 1.13 | 3.00 | 2.36 | 14.55 |
| aerobic_chemoheterotrophy | 108.88 | 109.71 | 12.45 | 218.10 |
| methanogenesis (disproportionation_of_methyl_groups) | 3.38 | 21.86 | 1.23 | 10.95 |
| nitrate_reduction | 2.25 | 8.43 | 9.32 | 118.50 |
| methylotrophy | 5.00 | 9.43 | 0.23 | 1.95 |
| aromatic_compound_degradation | 2.75 | 4.29 | 0.41 | 4.35 |
| sulfur_respiration | 6.25 | 0.00 | 0.00 | 0.00 |
| phototrophy | 0.00 | 0.00 | 0.00 | 8.35 |
| mammal_gut | 0.00 | 0.00 | 0.95 | 61.60 |
| aerobic_ammonia_oxidation | 0.00 | 0.00 | 0.00 | 3.25 |
| nitrification | 0.00 | 0.00 | 0.00 | 3.25 |
| photosynthetic_cyanobacteria | 0.00 | 0.00 | 0.00 | 6.85 |
| oxygenic_photoautotrophy | 0.00 | 0.00 | 0.00 | 6.85 |
| photoautotrophy | 0.00 | 0.00 | 0.00 | 6.85 |
| **A. Potential Microbial Functional Categories without *Blattabacteriaceae*** | **Ayayee_Embryo** | **Ayayee_Female_gut** | **Jennings _Embryo** | **Jennings _Female_gut** |
| hydrocarbon_degradation | 9.38 | 2.71 | 0.00 | 0.05 |
| ureolysis | 0.75 | 0.00 | 8.75 | 0.15 |
| sulfate_respiration | 1.75 | 3.00 | 2.25 | 8.60 |
| respiration_of_sulfur_compounds | 2.75 | 3.00 | 2.25 | 8.60 |
| aerobic_chemoheterotrophy | 14.25 | 3.29 | 19.25 | 8.00 |
| human_gut | 0.00 | 0.00 | 3.25 | 2.30 |
| mammal_gut | 0.00 | 0.00 | 3.25 | 2.35 |
| human_associated | 0.00 | 0.00 | 3.25 | 2.40 |
| human_pathogens_all | 0.00 | 0.00 | 0.50 | 0.15 |
| methanogenesis (reduction_of_methyl_compounds_with_H2) | 0.00 | 0.43 | 0.00 | 0.15 |
